# Association of a novel endometrial cancer biomarker panel with prognostic risk, platinum insensitivity, and targetable therapeutic options

**DOI:** 10.1371/journal.pone.0245664

**Published:** 2021-01-27

**Authors:** Jesus Gonzalez Bosquet, Qing Zhang, William A. Cliby, Jamie N. Bakkum-Gamez, Ling Cen, Sean C. Dowdy, Mark E. Sherman, S. John Weroha, Amy C. Clayton, Benjamin R. Kipp, Kevin C. Halling, Fergus J. Couch, Karl C. Podratz

**Affiliations:** 1 Department of Obstetrics and Gynecology, University of Iowa, Iowa City, Iowa, United States of America; 2 Division of Gynecologic Oncology, Mayo Clinic, Rochester, Minnesota, United States of America; 3 Moffitt Cancer Center, Tampa, Florida, United States of America; 4 Department of Health Sciences Research, Mayo Clinic, Jacksonville, Florida, United States of America; 5 Division of Medical Oncology, Mayo Clinic, Rochester, Minnesota, United States of America; 6 Department of Laboratory Medicine and Pathology, Mayo Clinic, Rochester, Minnesota, United States of America; Avera Research Institute, UNITED STATES

## Abstract

During the past decade, the age-adjusted mortality rate for endometrial cancer (EC) increased 1.9% annually with *TP53* mutant (*TP53*-mu) EC disproportionally represented in advanced disease and deaths. Therefore, we aimed to assess pivotal molecular parameters that differentiate clinical outcomes in high- and low-risk EC. Using the Cancer Genome Atlas, we analyzed EC specimens with available DNA sequences and quantitative gene-specific RNA expression data. After polymerase ɛ (*POLE*)-mutant specimens were excluded, differential gene-specific mutations and mRNA expressions were annotated and integrated. Consequent to *TP53*-mu failure to induce p21, derepression of multiple oncogenes harboring promoter p21 repressive sites was observed, including *CCNA2* and *FOXM1* (*P* < .001 compared with *TP53* wild type [*TP53*-wt]). *TP53*-wt EC with high *CCNA2* expression (*CCNA2*-H) had a targeted transcriptomic profile similar to that of *TP53*-mu EC, suggesting CCNA2 is a seminal determinant for both *TP53*-wt and *TP53*-mu EC. CCNA2 enhances E2F1 function, upregulating *FOXM1* and *CIP2A*, as observed in *TP53*-mu and *CCNA2*-H *TP53*-wt EC (*P* < .001). CIP2A inhibits protein phosphatase 2A, leading to AKT inactivation of GSK3β and restricted oncoprotein degradation; *PPP2R1A* and *FBXW7* mutations yield similar results. Upregulation of *FOXM1* and failed degradation of FOXM1 is evidenced by marked upregulation of multiple homologous recombination genes (*P* < .001). Integrating these molecular aberrations generated a molecular biomarker panel with significant prognostic discrimination (*P* = 5.8×10^−7^); adjusting for age, histology, grade, myometrial invasion, *TP53* status, and stage, only *CCNA2*-H/*E2F1*-H (*P* = .0003), *FBXW7*-mu/*PPP2R1A*-mu (*P* = .0002), and stage (*P* = .017) were significant. The generated prognostic molecular classification system identifies dissimilar signaling aberrations potentially amenable to targetable therapeutic options.

## Introduction

The American Cancer Society (ACS) predicted 61,880 new cases and 12,160 deaths that would be attributable to endometrial cancer (EC) in 2019 [1]. In 2018, the ACS reported an alarming 1.9% annual increase during the decade in age-adjusted mortality for EC [2]—a trajectory needing reversal. Standard treatment for high-risk EC is definitive surgery followed by systemic platinum-based chemotherapy (PbCT) or radiotherapy, or both. Sensitivity to PbCT positively correlates with deficiencies in the homologous recombination (HR) pathway [3]. However, the majority of ECs are HR proficient; thus, tailored molecular-based therapy needs to be developed, which requires identifying molecular profiles that harbor targetable aberrations.

The clinical outcomes associated with *TP53* mutated (*TP53*-mutant [mu]) EC are strikingly worse than those observed with *POLE* mutations (exonuclease domain of polymerase ɛ mutant, catalytic subunit [*POLE*-mu]) and *TP53* wild type (*TP53*-wt) tumors [4]. The tumor suppressor functions of TP53 include transcription activation and repression; exemplary of the former is the activation of *CDKN1A*, encoding p21, which targets promoter-repressive elements (cell-cycle–dependent elements [CDE] and cell-cycle genes homology region [CHR] sites), resulting in transcription repression of targeted genes [5]. Mutant *TP53* is unable to activate the TP53-p21-CDE/CHR axis. Thus, *TP53*-mu cancers have derepression of numerous genes containing promoter CDE/CHR sites, including *CDK2*, *CCNA2*, *AURKA*, *TPX2*, *PLK1*, *FOXM1*, *MASTL*, and *ESPL1* [5].

Upregulated CDK2 phosphorylates pRB, releasing pRB-bound E2F1, a potent transcription activator [6]. Mints et al [7] reported progressively increasing nuclear expression of E2F1 with decreasing differentiation of EC. The E2F1 mode of action is predicated on CCNA2 expression; overexpression of CCNA2 has been correlated with compromised prognosis and resistance to chemotherapy in EC [8–10]. *CCNE1*, *AURKA*, *TPX2*, *PLK1*, *FOXM1*, *EZH2*, *CIP2A*, *BRCA1*, and *RAD51* have E2F1 activation sites in their promoter regions [11–14]. E2F1 activation of critical genes portends increased phosphorylation of the cohesion complex with premature chromosome separation (ie, aneuploidy) as well as FOXM1 induction of several genes in the HR pathway [15–18].

The Cancer Genome Atlas (TCGA) for EC documented the high prevalence of *PIK3CA*, *PIK3R1*, *PTEN*, *PPP2R1A*, and *FBXW7* mutations, genes within the PI3K-AKT-FBW7 axis [4]. Mutations in *PIK3CA*, *PIK3R1*, and *PTEN* facilitate the phosphorylation and activation of AKT, which phosphorylates and inactivates GSK3β resulting in restricted FBW7-dependent degradation of oncoproteins such as CCNE1, AURKA, PLK1, FOXM1, and others [19]. AKT activation is modulated by protein phosphatase 2A (PP2A), but mutations in its subunit (*PPP2R1A*) or upregulation of its endogenous inhibitor (CIP2A) allow unimpeded AKT phosphorylation [20, 21]. *CIP2A* is reportedly activated by E2F1 [14]. Thus, AKT inactivation of GSK3β or mutation in *PPP2R1A* or *FBXW7* results in restricted degradation and accumulation of specific oncoproteins.

**Fig 1:**
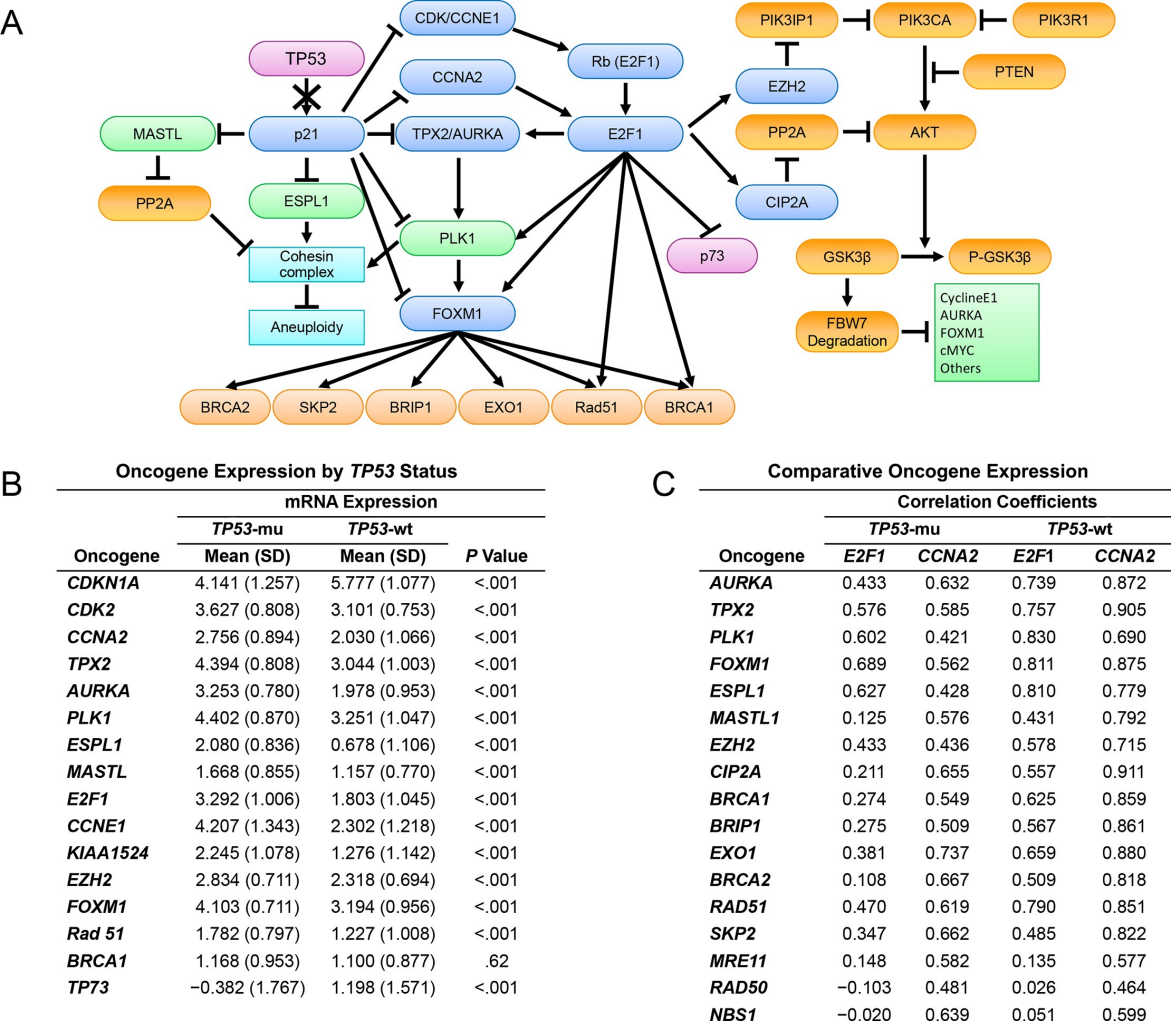
Molecular schematic and oncogene expression. A, Integrated schematic of the TP53-p21-CDE/CHR and PIK3CA-AKT-FBW7 pathways in EC. B, Comparative analysis of the mRNA expression of oncogenes regulated directly or indirectly by the TP53-p21-CDE/CHR pathway in *TP53* mutant (mu) (n = 62) vs wild type (wt) EC (n = 149) (excluding *POLE* mutants). C, Correlation coefficient analysis delineating relationships between differentially expressed oncogenes in *TP53*-mu (n = 62) and wt EC (n = 149) (excluding *POLE* mutants) as a function of mRNA expression of *E2F1* and *CCNA2* oncogenes. CDE indicates cell-cycle–dependent element; CHR, cell-cycle genes homology region; EC, endometrial cancer; wt, wild type.

Integrating the above generic TP53 mechanistic information with data available from the EC literature, we developed a working schematic (Fig 1A) for comparing the mRNA expression between *TP53*-mu and *TP53*-wt EC for numerous genes that impact cell-cycle dynamics, apoptosis, and DNA-damage repair. We identified the seminal role of CCNA2 in 1) integrating the TP53-p21-CDE/CHR and PI3K-AKT-FBW7 pathways and 2) combining with *E2F1* overexpression and mutations in *FBXW7* and *PPP2R1A* in determining outcomes of both *TP53*-mu and *TP53*-wt EC. An untoward commonality included induction of *FOXM1* or failed degradation of FOXM1, or both, which portends enhanced HR gene expression and potential insensitivity to chemotherapy.

## Methods

### The Cancer Genome Atlas

We obtained and analyzed TCGA (www.cancergenome.nih.gov) data as previously described [22]. TCGA contains comprehensive genomic information including copy number variation, single-nucleotide polymorphisms, miRNA expression, gene expression, and DNA methylation data, as well as clinical and outcome information. Data from TCGA were downloaded, normalized, formatted, and organized for integration and analysis with other biological datasets in accordance with TCGA data-sharing agreements. Somatic mutations and gene expression data were recorded.

All data collection and processing, including the consenting process, were performed after approval by each of the participating institution’s local institutional review board/ethics committee and in accordance with TCGA Human Subjects Protection and Data Access Policies, adopted by the National Cancer Institute and the National Human Genome Research Institute.

### Mutation analysis

Somatic mutation detection, calling, annotation, and validation from TCGA have been described [23]. Somatic mutation information resulting from exome sequencing with the Illumina Genome Analyzer DNA Sequencing GAIIx or HiSeq 2000 platforms (Illumina Inc) was downloaded and formatted for analysis. Mutation information was downloaded as level 3 or validated somatic mutations.

Of the 239 cancerous endometrial tumors included, we identified 18,388 unique genes with 138,838 validated somatic mutations, including frame-shift insertions and deletions; in-frame insertions or deletions; and missense, nonsense, nonstop, and splice-site mutations. Silent mutations were excluded from the analysis. The number of mutations for each selected gene was recorded for each patient.

### Gene expression

Gene expression data were downloaded from TCGA data repository as level 3 RNA sequence data [4] created by Illumina RNA Sequencer HiSeq 2000 platforms (Illumina Inc) and annotated with the HG-19 version of the human genome. Normalized and log-transformed gene expression data from these endometrial tumors were available for analysis. Analyses were performed with R statistical packages (R Foundation) for statistical computing and graphics [24] and bioconductor packages as open-source software for bioinformatics [25]. For the front end, we used Biometric Research Branch Array Tools, an integrated package for visualization and statistical analysis that uses Excel (Microsoft Corp) [26].

### Cell lines and in vitro assessments

As PbCT is the predominant adjuvant therapy for high-risk EC, which are frequently insensitive to therapy [2, 27–30], we chose cell lines recognized as platinum insensitive with identified mutational anomalies associated with adverse clinical outcomes in the study population. ARK2, a uterine serous carcinoma (USC) (type II) derived cell line, harbors mutant *TP53* and wt *FBXW7* and *PPP2R1A* (personal communication with A. Santin, Yale University) [31]. HEC-1B cells (endometrioid endometrial carcinomas [EEC]; type I) have mutations in *TP53*, *FBXW7*, and *PPP2R1A* [32]. Both cell lines were cultured in Dulbecco’s Modified Eagle’s Medium containing 10% fetal bovine serum, 100 mcg/mL streptomycin, 100 units/mL penicillin, and 2 mM L-glutamine. Cells were maintained in an incubator at 37°C in an atmosphere containing 5% CO_2_. Carboplatin and panobinostat (HDAC10 inhibitor) were purchased from ApexBio.

### Real-time polymerase chain reaction

Total RNA was isolated using RNeasy Plus MiniK (Qiagen). cDNA was synthesized using a Reverse Transcription Kit (Applied Biosystems). Real-time polymerase chain reaction (PCR) was performed using the SYBR Green PCR Master Mix (ThermoFisher Scientific) on the LightCycler 480 (Roche Molecular Systems Inc). The sequences of primers for the analyzed genes are detailed in S1 Table.

### Western blot analysis

ARK-2 cells were treated with panobinostat at 10 nM. After incubation for 3, 6, 12, and 24 hours, cell lysates were collected for protein expression analyses and compared with untreated (time = 0) controls. Expression of p21, FOXM1, acetylated-H3, and GAPDH were measured by Western blot. Antibodies used in this study were P21 (Cell Signaling Technology, 2947), FOXM1 (Cell Signaling Technology, 5436), acetyl-H3 (Millipore, 06–599), and GAPDH (Sigma-Aldrich, G8795).

### MTT assay and synergy assessment

Three thousand cells per well were seeded in triplicate in 96-well plates and the cells treated with increasing concentrations of panobinostat and carboplatin for 72 hours, respectively. MTT-based CellTiter 96 Aqueous One Solution Cell Proliferation Assay (Promega Corp) was performed (per manual) to assess half-maximum inhibitory concentration. Constant-ratio studies were performed to investigate the combinatory effect of carboplatin with panobinostat in HEC-1B and ARK-2 cell lines [33].

### Statistical analysis

For each candidate gene surveyed, TCGA-quantitated expression levels of the corresponding mRNA were annotated for the 239 specimens. Comparisons between groups were evaluated with the χ^2^ test for nominal variables and the 2-sample *t* test for continuous variables. Correlations were quantified by using Pearson correlation coefficients. All calculated *P* values were 2-sided.

### Progression-free survival analysis

Statistical methods for survival data were used to analyze progression-free survival (PFS), defined as the time from surgery to disease recurrence. Patients without evidence of disease at the end of follow-up were treated as censored observations. Comparisons between Kaplan-Meier survival curves were performed with log-rank tests. For association with survival, all clinicopathologic variables were assessed with Cox proportional hazard regression. All variables associated with survival with a univariate *P* value ≤.05 were included in an initial multivariate regression model. Those variables with the smallest contributory effect were excluded with a backward elimination technique based on the Akaike information criterion (measure of the quality of the model for a given dataset). Hazard ratios (95% CI) were reported. Analyses were performed using R statistical computing and graphics [24].

## Results

### Study tumor characteristics

Using TCGA for EC, we analyzed specimens with available DNA sequences and quantitative gene-specific RNA expression data [4]. Clinicopathologic characteristics of the tumors in the study population (N = 239) included 47 (26, stage 3/4) USC and 192 EEC including 72 grade 1 (4, stage 3/4), 73 grade 2 (10, stage 3/4), and 47 grade 3 (16, stage 3/4). Molecular characteristics included *POLE*-mu detected in 28 specimens (11.7%), *TP53*-mu in 70 (29.3%), microsatellite instability-high (MSI-H) in 67 (28%), and estimated copy number variation low (CNV-L) (determined by 239 –[*POLE*-mu + *TP53*-mu + MSI-H]) in 92 (38.5%). *TP53*-mu was identified in 41 (87.2%) USC and 29 (15.1%) EEC including 4.2% in grade 1, 12.3% in grade 2, and 36.5% in grade 3. At least 1 mutation in *PIK3CA*, *PTEN*, or *PIK3R1* occurred in 83% of the specimens; 57% had a mutation in more than 1 of these genes. Because *POLE*-mu was associated with ultramutated status and superior outcomes [4], *POLE* mutants were not included in the subsequent molecular analyses except to use as a standard for comparing favorable outcomes. Thus, the primary study population consisted of 62 *TP53*-mu and 149 *TP53*-wt specimens. Of note, compared with the general EC population, the study population was weighted toward more high-risk characteristics, as shown by the enhanced prevalence of advanced disease, grade 3 histology, and USC.

### Comparative assessment of oncogene expression in *TP53*-mu and *TP53*-wt

To assess the validity of the proposed downstream network of *TP53*-mu–dependent gene alterations in Fig 1A, we compared the mean mRNA expression level of the proposed gene network in *TP53*-mu and *TP53*-wt EC, excluding *POLE*-mu specimens. Assessment of *CDKN1A* (p21) expression in *TP53*-mu compared with that in *TP53*-wt EC showed a dramatic differential consistent with the failure of mutated TP53 to induce *CDKN1A* (p21) (Fig 1B). Multiple genes harboring CDE/CHR p21 repressive site in their promoter regions, including *CDK2*, *CCNA2*, *AURKA*, *TPX2*, *PLK1*, *FOXM1*, *ESPL1*, and *MASTL* [5], were significantly upregulated in *TP53*-mu compared with *TP53*-wt EC. The lack of suppression of CDK2 and marked overexpression of *CCNE1* and *E2F1* portend the observed augmentation of multiple cell cycle (ie, *AURKA*, *TPX2*, *PLK1*) and other genes (ie, *FOXM1*, *Rad51*, and *CIP2A* [formerly *KIAA1524*]) harboring E2F1 transcriptional activating sites [11–14]. By contrast, the E2F1 apoptotic target *TP73* is significantly suppressed in *TP53*-mu tumors [8].

### Oncogene expression correlation with CCNA2 and E2F1

The overexpression of *E2F1* and concomitant *TP73* suppression in *TP53*-mu EC suggested, as previously reported, upregulation of CCNA2, which determines the mode of action of E2F1 [8, 34]. Thus, we examined the correlation between reference oncogenes (*E2F1* and *CCNA2*) and multiple direct or downstream targets of E2F1 in *TP53*-mu and *TP53*-wt EC (Fig 1C). Correlation coefficients for the reference genes in *TP53*-mu tumors were similarly positive with regard to cell-cycle genes, but the positivity was substantially higher for *CCNA2* than *E2F1* for *MASTL1*, *CIP2A*, and HR pathway genes. Unexpected were the high positive correlations in *TP53*-wt tumors between the expressions of *CCNA2* and E2F1 targets and HR pathway genes, which paralleled the correlations in *TP53*-mu tumors. These results suggested a potential role for CCNA2 in the carcinogenesis of both *TP53*-mu and a subset of *TP53*-wt tumors.

### Comparative expression of oncogenes as a function of *TP53*-mu and *TP53*-wt CCNA2 expression

The expression of multiple, upregulated oncogenes in *TP53*-mu EC was assessed in *TP53*-wt EC with high *CCNA2* expression. The upper quartile of annotated CCNA2 mRNA expression levels among *TP53*-wt specimens (≥2.6) was arbitrarily designated as high expression (*CCNA2*-H). When the expression of multiple CCNA2/E2F1 target and HR-pathway genes in *TP53*-wt *CCNA2*-H and *TP53*-mu EC was assessed, equivalency or higher expression was shown for most assessed genes in *TP53*-wt *CCNA2*-H vs *TP53*-mu specimens (Table 1). Noteworthy was the dramatic upregulation of *FOXM1*, *CIP2A*, and multiple HR genes in both *TP53*-mu and *TP53*-wt *CCNA2*-H EC compared with *TP53*-wt with CCNA2 low expression (*CCNA2*-L).

**Table 1:** Comparison of the expression of multiple pathway-specific genes in *TP53* wild type/*CCNA2*-high vs *TP53* mutant vs *TP53* wild type/*CCNA2*-low specimens.

	Cohort A	Cohort B	Cohort C	Cohort A vs B		Cohort B vs C	
	*TP53* Mutants	*CCNA2*-High	*CCNA2*-Low				
	(n = 62)	(n = 41)	(n = 108)				
Gene	Mean (SD)	Mean (SD)	Mean (SD)	Cohen’s d^a^	*P* Value	Cohen’s d^a^	*P* Value
**TP53-p21-CDK2-E2F1/CCNA2 Pathway**							
*CDKN1A*	4.141 (1.257)	5.648 (1.070)	5.826 (1.080)	1.271	< .001	0.165	.37
*CDK2*	3.627 (0.808)	3.867 (0.529)	2.811 (0.608)	0.337	.10	1.797	< .001
*E2F1*	3.292 (1.006)	2.703 (0.938)	1.462 (0.868)	0.602	.004	1.398	< .001
*CCNA2*	2.756 (0.894)	3.276 (0.569)	1.557 (0.793)	0.665	.001	…	…
**E2F1/CCNA2 Targets**							
*CCNE1*	4.207 (1.343)	2.876 (1.085)	2.084 (1.199)	1.067	< .001	0.678	< .001
*AURKA*	3.253 (0.780)	2.920 (0.738)	1.621 (0.765)	0.436	.03	1.715	< .001
*TPX2*	4.394 (0.808)	4.117 (0.654)	2.637 (0.791)	0.369	.07	1.957	< .001
*PLK1*	4.402 (0.870)	4.234 (0.811)	2.877 (0.872)	0.198	.33	1.585	< .001
*FOXM1*	4.103 (0.800)	4.229 (0.653)	2.801 (0.733)	0.169	.40	2.003	< .001
*EZH2*	2.834 (0.711)	2.898 (0.528)	2.098 (0.620)	0.098	.63	1.340	< .001
*CIP2A*	2.245 (1.078)	2.435 (0.690)	0.835 (0.956)	0.201	.32	1.794	< .001
**Homologous Recombination Pathway Genes**							
*MER11*	0.975 (0.885)	1.264 (0.537)	0.624 (0.755)	0.377	.06	0.912	< .001
*RAD50*	1.940 (0.753)	2.461 (0.680)	2.021 (0.603)	0.719	< .001	0.704	< .001
*NBS1*	2.473 (0.987)	2.987 (0.644)	2.340 (0.836)	0.593	.004	0.820	< .001
*BRCA1*	1.168 (0.953)	1.947 (0.744)	0.778 (0.691)	0.889	< .001	1.656	< .001
*BRIP1*	−0.354 (1.027)	0.276 (0.805)	−1.151 (0.808)	0.667	.001	1.767	< .001
*EXO1*	1.092 (0.919)	1.509 (0.705)	0.023 (1.027)	0.496	.02	1.563	< .001
*BRCA2*	−1.146 (1.355)	−0.395 (0.877)	−2.310 (1.200)	0.631	.002	1.708	< .001
*RAD51*	1.782 (0.797)	2.275 (0.679)	0.829 (0.807)	0.655	.002	1.867	< .001

^a^ Cohen’s d = the absolute value of the difference in group means divided by the pooled standard deviation; the higher the value, the greater the difference between groups: ≥0.2/<0.5, small; ≥0.5/<0.8, medium; and ≥0.8, large.

### Clinical outcomes according to EC classifications

**Fig 2:**
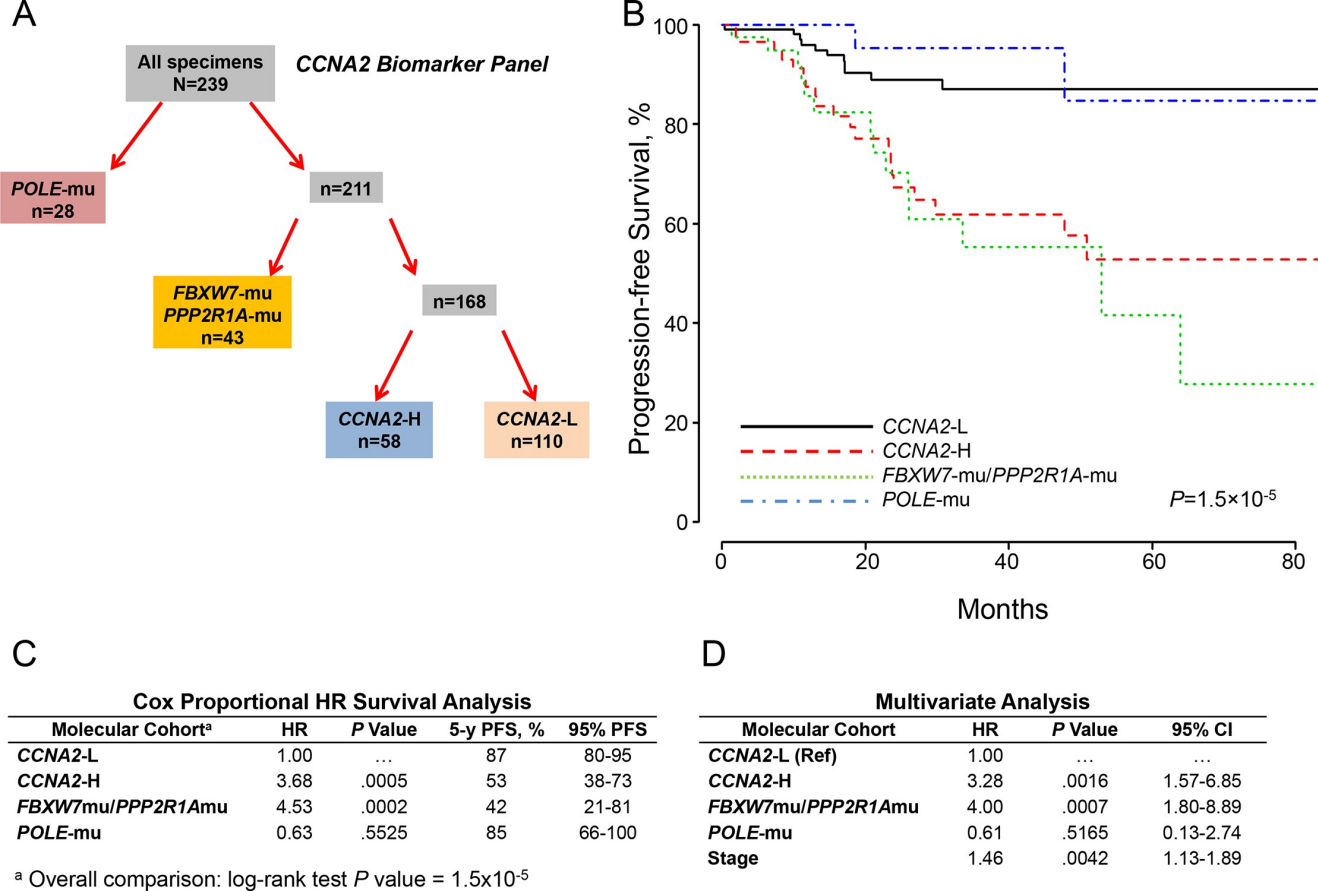
Molecular classification by cohorts and outcomes (*CCNA2* expression). A, Molecular classification differentiated 4 cohorts according to *POLE* mutations (*POLE*-mu), *FBXW7* and/or *PPP2R1A* mutations (*FBXW7*-mu/*PPP2R1A*-mu), high *CCNA2* expression (*CCNA2*-H), and low *CCNA2* expression (*CCNA2*-L). B, PFS as a function of time according to molecular cohorts. C, Cox proportional model analysis of the molecular classification cohorts using *CCNA2*-L as the reference. D, Multivariate analysis including the configured panel cohorts, age, grade, histology, myometrial invasion, *TP53* status, and stage. HR indicates hazard ratio; PFS, progression-free survival.

The molecular schematic suggested that the high expression of *FOXM1* observed with upregulated *CCNA2* expression in *TP53*-mu and *TP53*-wt (Fig 1A), combined with anticipated restricted proteosomal degradation of FOXM1 due to *PPP2R1A*-mu or *FBXW7*-mu, would unfavorably impact survival [35]. Accordingly, the study population was segregated into 4 cohorts including *POLE*-mu, *PPP2R1A*-mu/*FBXW7*-mu, *CCNA2*-H, and *CCNA2*-L (Fig 2A). PFS analysis for *POLE* mutants was as previously reported [4], but the *CCNA2*-H and *PPP2R1A*-mu/*FBXW7*-mu cohorts had substantially disparate outcomes compared to the *CCNA2*-L cohort (Fig 2B). Cox proportional hazard ratio survival analysis using *CCNA2*-L as the reference assigned significance for *CCNA2*-H (hazard ratio, 3.68; *P* = .0005) and *PPP2R1A*-mu/*FBXW7*-mu (hazard ratio, 4.53; *P* = .0002) (Fig 2C). Adjusting for age, histology, grade, myometrial invasion, *TP53*-mu status, and stage, independent significance (PFS) was associated with *CCNA2*-H (*P* = .0016), *PPP2R1A*-mu/*FBXW7*-mu (*P* = .0007), and stage (*P* = .0042) (Fig 2D).

**Fig 3:**
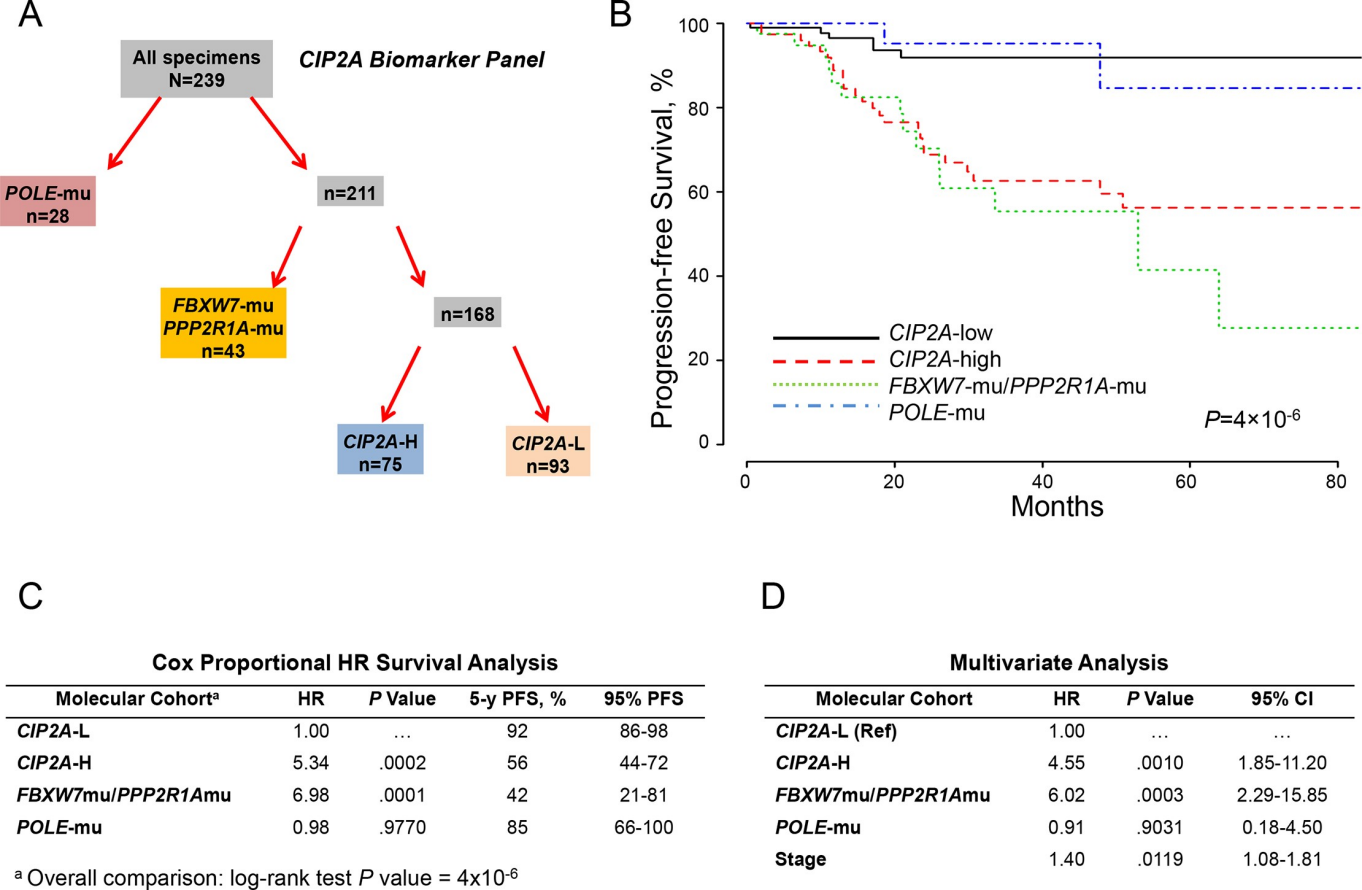
Molecular classification by cohorts and outcomes (*CIP2A* expression). A, Molecular classification differentiated 4 cohorts according to *POLE* mutations (*POLE*-mu), *FBXW7* and/or *PPP2R1A* mutations (*FBXW7*-mu/*PPP2R1A*-mu), high *CIP2A* expression (*CIP2A*-H), and low *CIP2A* expression (*CIP2A*-L). B, PFS as a function of time according to molecular cohorts. C, Cox proportional model analysis of the molecular classification cohorts using *CIP2A*-L as the reference. D, Multivariate analysis including the configured panel cohorts, age, grade, histology, myometrial invasion, *TP53* and *PTEN* mutational status, and stage. HR indicates hazard ratio; PFS, progression-free survival.

Considering E2F1 activates CIP2A [14], which modulates the PI3K-AKT-FBW7 axis via inhibition of PP2A [20, 21], we replaced *CCNA2* expression with *CIP2A* expression (*CCNA2*:*CIP2A* correlation coefficient, 0.893). Stratifying the molecular panel into *POLE*-mu, *PPP2R1A*-mu/*FBXW7*-mu, *CIP2A*-H, and *CIP2A*-L (Fig 3A) produced correspondingly significant discriminatory outcomes (Fig 3B), as judged by Cox proportional hazard ratios of 5.34 and 6.98 for *CIP2A*-H and *PPP2R1A*-mu/*FBXW7*-mu, respectively (Fig 3C). *CIP2A* overexpression and *PPP2R1A*-mu would portend PP2A deficiency and unimpeded activation of AKT, the latter potentially augmented by upstream dysregulated elements that initiate the PI3K-AKT kinase cascade [4, 19–21, 36, 37]. Therefore, mutant *PTEN*, *PIK3CA*, *PIK3R1*, and *ARID1A* and *ERBB2* expression were included in the univariate analysis; only *PTEN*-mu was significant. Adjusting for age, grade, histology, myometrial invasion, *TP53* and *PTEN* mutational status, and stage, independent significance was associated with *CIP2A*-H-mu (*P* = .001), *PPP2R1A*-mu/*FBXW7*-mu (*P* = .0003), and stage (*P* = .0119) (Fig 3D).

**Fig 4:**
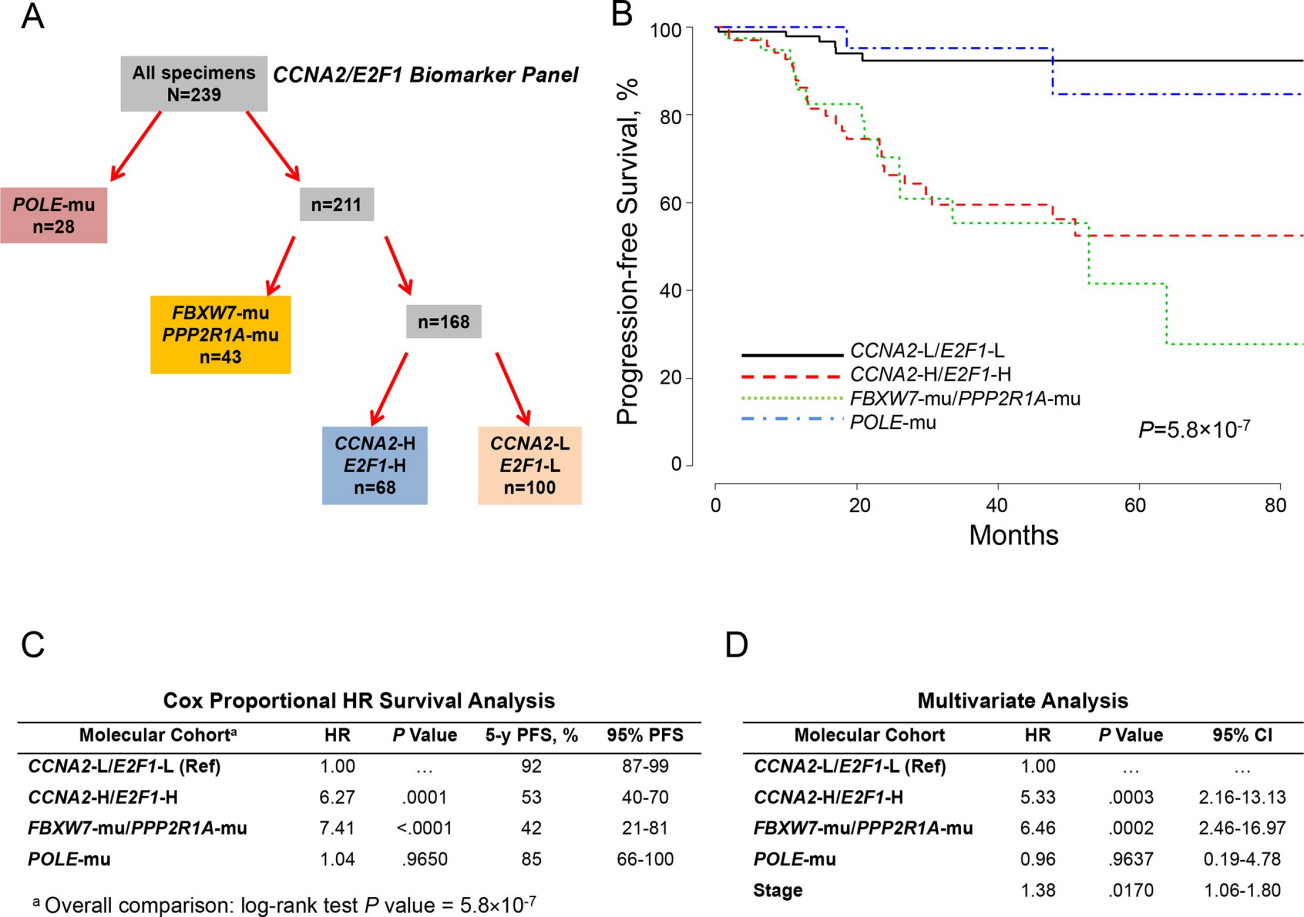
Molecular classification by cohorts and outcomes (*CCNA2/E2F1* Expression). A, Molecular classification differentiated 4 cohorts according to *POLE* mutations (*POLE*-mu), *FBXW7* and/or *PPP2R1A* mutations (*FBXW7*-mu/*PPP2R1A*-mu/), high *CCNA2* and *E2F1* expression (*CCNA2*-H/*E2F1*-H), and low *CCNA2* and *E2F1* expression (*CCNA2*-L/*E2F1*-L). B, PFS as a function of time according to molecular cohorts. C, Cox proportional model analysis of the molecular classification cohorts using *CCNA2*-L/*E2F1*-L as the reference. D, Multivariate analysis including the configured panel, age, grade, histology, myometrial invasion, *TP53* status, and stage. HR indicates hazard ratio; PFS, progression-free survival; Ref, reference.

Recognizing the seminal role of CCNA2 in regulating E2F1 and indirectly CIP2A and FOXM1 in both *TP53*-mu and *TP53*-wt EC, we postulated that high expression of either *CCNA2* or *E2F1* with more modest expression of the other would further discriminate outcomes. Slightly more restrictive levels for *CCNA2* (≥2.75) and *E2F1* (≥2.75) expression were used. This allowed stratifying EC into 4 molecular-based distinguishable cohorts (Fig 4A) associated with distinct, long-term PFS outcomes (Fig 4B). Using the low-expression cohort for *CCNA2* and *E2F1* (*CCNA2*-L/*E2F1*-L) as reference, Cox proportional survival analysis showed significant hazard ratios for the *FBXW7*-mu/*PPP2R1A*-mu and *CCNA2*-H/*E2F1*-H cohorts (Fig 4C). Adjusting for age, grade, histology, myometrial invasion, stage, and *TP53* status, Cox analysis showed independent significance for *CCNA2*-H/*E2F1*-H (hazard ratio, 5.33; *P* = .0003), *FBXW7*-mu/*PPP2R1A*-mu (hazard ratio, 6.46; *P* = .0002), and stage (hazard ratio, 1.38; *P* = .0170) (Fig 4D).

### Recurrences in traditional low-risk and high-risk EC according to biomarker panel cohorts

Contemporary adjuvant therapy for low-risk EC (stage 1 or 2, grade 1 or 2) is generally limited. These low-risk tumors significantly (*P* < .0001) stratified according to molecular-panel cohorts. The estimated 5-year PFS for low-risk EC with the low-risk biomarker profile (*CCNA2*-L/*E2F1*-L/*FBXW7*-wt/*PPP2R1A*-wt) (n = 75) was 92% compared with 31% for the low-risk EC with the high-risk biomarker profile (*CCNA2*-H/*E2F1*-H or *FBXW7*-mu/*PPP2R1A*-mu, or both) (n = 35) (S1 Fig). By contrast high-risk EC (stage 3 or 4 and/or grade 3) are frequently managed with adjuvant PbCT. Stratified by biomarker panel profiles, high-risk patients with the low-risk biomarker profile (n = 25) appeared to respond favorably to contemporary therapy (estimated 5-year PFS, 93%) compared with those who had the high-risk biomarker profile (n = 76) (estimated 5-year PFS, 56%) (*P* = .023) (S1 Fig).

### Clinical outcomes according to biomarkers in MSI-H and CNV-L EC

Considering the reported emphasis on MSI-H and CNV-L in TCGA for EC [4], we assessed PFS associated with MSI-H (excluding *POLE*-mu) and CNV-L for *CCNA2*-L/*E2F1*-L/*FBXW7*-wt/*PPP2R1A*-wt vs *CCNA2*-H/*E2F1*-H or *FBXW7*-mu/*PPP2R1A*-mu, or a combination. The biomarker panel cohorts separate both MSI-H (estimated 5-year PFS, 95% and 42%, respectively) and CNV-L (estimated 3-year PFS, 92% and <50%, respectively) into 2 diverse prognostic subgroups (S2 and S3 Figs, respectively), suggesting an inclusive applicability for the molecular biomarker classification panel.

### HR pathway gene expression as a function of adverse biomarkers

*FOXM1* transcription was dramatically increased in the *CCNA2*-H/*E2F1*-H cohort compared with the *CCNA2*-L/*E2F1*-L cohort. Moreover, the expression of *CIP2A* (formerly *KIAA1524*) and the genes in the HR pathway (*EXO1*, *BRIP1*, *Rad51*, *BRCA1*, and *BRCA2*) reportedly induced by FOXM1 [18] were significantly upregulated in both the *CCNA2*-H/*E2F1*-H and *FBXW7*-mu/*PPP2R1A*-mu cohorts compared with the *CCNA2*-L/*E2F1*-L cohort (S2 Table).

### Induction of p21 and repression of panel-specific targets

**Fig 5:**
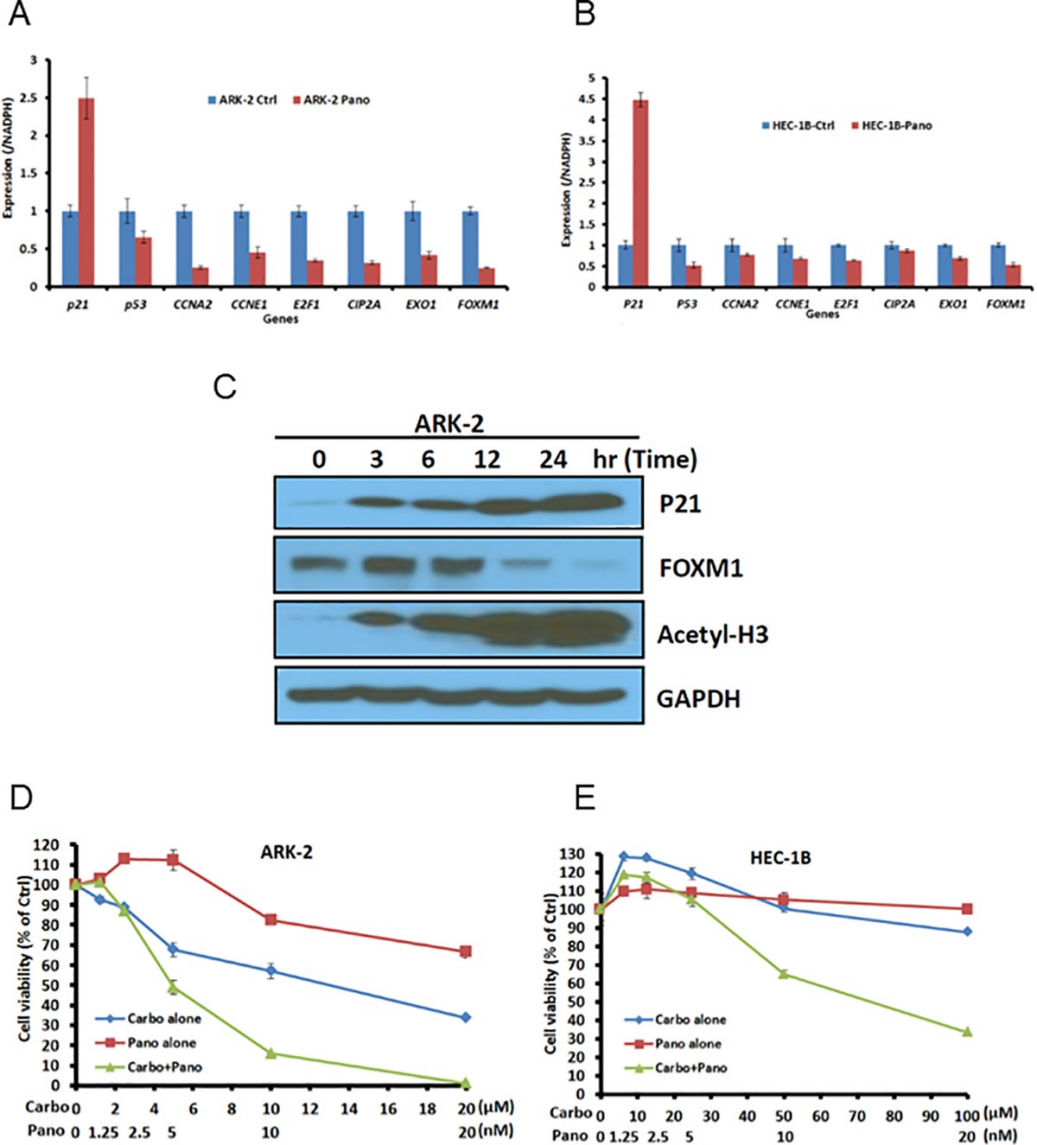
In vitro assessment of ARK-2 and HEC-1B cell line response to HDAC inhibitor. A and B, After 24-hour exposure to panobinostat or vehicle, target-specific gene expression was determined by quantitative polymerase chain reaction in ARK-2 and HEC-1B cell lines. C, ARK-2 cells untreated or treated with 10 nM panobinostat and protein expression assessed at indicated time points (Western blot). D and E, Cell viability assays in ARK-2 and HEC-1B cell lines after exposure to panobinostat or carboplatin alone and in combination, assessed to determine synergism. Carbo indicates carboplatin; ctrl, control; pano, panobinostat.

The molecular schematic (Fig 1A) predicts that *CDKN1A* (p21) induction in *TP53*-mu tumors would repress multiple oncogenes with downstream suppression of corresponding targets. Histone deacetylase inhibitors (HDACi) have been reported to induce p21 in *TP53*-mu cell lines [38]. The platinum-insensitive cell lines ARK-2 and HEC-1B were exposed to panobinostat, an HDAC10 inhibitor, and qPCR expression of targeted genes analyzed. Increased expression of *CDKN1A* (p21) with downregulation of *CCNA2*, *E2F1*, *CIP2A*, *FOXM1*, and *EXO1* was observed in both cell lines (Fig 5A and 5B).

ARK-2 cells were treated with 10 nM panobinostat and protein expression assessed via Western blot. Increased expression of p21 and acetyl-H3 and down-regulation of FOXM1 expression occurred in a time-dependent manner (Fig 5C). The stimulatory effect on p21 and the inhibitory effect of FOXM1 expression in response to panobinostat are consistent with the results observed in real-time PCR analysis (Fig 5A).

### Synergism with HDACi and carboplatin in platinum-insensitive cell lines

The downregulation of *FOXM1* and HR pathway *EXO1* with panobinostat in platinum-insensitive cell lines suggested the potential for HDACi to enhance platinum sensitivity. Synergism occurred in ARK-2 and HEC-1B cell lines exposed to varying concentrations of carboplatin and panobinostat (Fig 5D and 5E). These observations suggested that suppression of FOXM1 and HR pathway components might enhance platinum sensitivity in high-risk HR-proficient EC.

## Discussion

To our knowledge, this is the first report of a classification system for EC that appears to correlate with oncologic outcomes independent of patient age, histology, tumor grade, myometrial invasion, and *TP53* mutational status. The discriminatory PFS value of the cohorts in the molecular biomarker panel was predicated on the overexpression of *CCNA2* and *E2F1* or mutations in *FBXW7* or *PPP2R1A*. These observations constitute a mechanistic commonality regardless of *TP53* status that is equally applicable in MSI-H and CNV-L cohorts. Pivotal is the interactive role of CCNA2 and E2F1 in upregulating *FOXM1* transcription and inducing CIP2A activation, predictably leading to PP2A inhibition and likely restriction of FOXM1 degradation [19–21, 35, 39, 40]. The latter is likewise anticipated with *FBXW7* and *PPP2R1A* mutations. FOXM1 reportedly induces multiple HR genes such as *BRIP1*, *BRCA2*, *EXO1*, and *Rad51* [18], all of which were overexpressed in the poor prognostic molecular biomarker cohorts. The mechanistic molecular distillate from our observations suggests that the overexpression of multiple HR-pathway genes expectedly limits responses in the majority of HR-proficient ECs treated with DNA-damaging agents.

The 1.9% annual increase in age-adjusted mortality for EC observed over the past decade warrants reappraisal of contemporary therapeutic algorithms [2]. Our recent institutional assessments coupled with subgroup analyses in select randomized clinical trials suggest that PbCT has suboptimal efficacy for managing high-risk EC [27–30]. Considering that most EC is HR proficient [4], augmenting HR components, several of which are induced by FOXM1, would presumably enhance DNA-damage repair, yielding insensitivity to DNA-damaging agents such as platinum [18]. This study confirms the marked upregulation of HR components in high-risk EC.

The integrated signaling pathways shown in Fig 1 illustrate the mechanisms that lead to simultaneous upregulation of *FOXM1* and downregulation of FOXM1 degradation in *TP53*-mu and *TP53*-wt with *CCNA2*-H and/or *E2F1*-H. The failure of TP53-mu to induce *CDKN1A* (p21) derepresses *FOXM1*, and with the upregulation of E2F1, *FOXM1* expression is further augmented [18]. The mechanism of action of E2F1 is predicated on CCNA2; high CCNA2 projects a proliferative E2F1 mode [8]. *E2F1* and *CCNA2* were both upregulated in *TP53*-mu and a subset of *TP53*-wt EC. Overexpression of CCNA2 has previously been correlated with TP53 expression, chemoresistance, and poor prognosis in EC [9, 10]. We showed for the first time that *TP53*-wt EC with high *CCNA2* expression is associated with molecular aberrations and clinical outcomes similar to *TP53*-mu EC. The mechanism responsible for high expression of *CCNA2* in the subset of *TP53*-wt EC is unknown.

The prognostic biomarker panel that incorporates *CCNA2*/*E2F1* upregulation and *PPP2R1A*/*FBXW7* mutations is highly discriminatory. Without these molecular aberrations, clinical outcomes are very favorable and appear to be similar to those of *POLE*-mu tumors. Importantly, the majority of EC is HR proficient, which predicts a high prevalence of platinum insensitivity in biomarker panel–positive patients. Suppressing the induction of *FOXM1* or enhancing degradation of FOXM1, or both, thereby downregulating HR components, might potentially facilitate conversion to platinum sensitivity. Exemplary exposure of platinum-insensitive *TP53*-mu EC cell lines to panobinostat [38], an HDACi currently in clinical trials, resulted in induction of *CDKN1A* (p21), suppression of *CCNA2*, *CIP2A*, *FOXM1*, and *EXO1*, and synergism with carboplatin at nM levels of panobinostat.

The strengths of this study include the robustness of TCGA annotated database, which includes specimens obtained at cancer centers dedicated to definitive management of patients with EC. The study is limited by the lack of biomarker-panel validation in a similar, sizeable population having definitive staging, central pathology review, standardized treatment, extended surveillance, and focused molecular analysis. The unavailability of detailed treatment algorithms and reliable long-term disease-specific survival documentation limited correlations of molecular irregularities to PFS and clinicopathologic parameters.

In summary, the integration of *CCNA2* and *E2F1* overexpression and *POLE*, *PPP2R1A* and *FBXW7* mutations generated a molecular EC classification that projects prognostic risk, platinum insensitivity, and potential targetable therapeutic options.

## Supporting information

S1 Fig Survival for low- and high-risk endometrial cancer according to molecular cohorts. (JPG)

S2 Fig Survival in MSI high endometrial cancer according to molecular cohorts. (JPG)

S3 Fig Survival in copy number variation low endometrial cancer according to molecular cohorts. (JPG)

S1 Table PCR primer sequences. (DOCX)

S2 Table Gene expressions as a function of molecular panel cohorts. (DOCX)

## Abbreviations

ACS: American Cancer Society
AURKA: aurora kinase A
CCNA2: cyclin A2
*CCNA2*-L: *CCNA2* low expression
*CCNA2*-H: *CCNA2* high expression
CDE: cell-cycle–dependent element
CDK2: cyclin-dependent kinase
CHR: cell-cycle genes homology region
CIP2A: cancerous inhibitor of protein phosphatase 2A
CNV-L: copy number variation low
EC: endometrial cancer
EEC: endometrioid endometrial carcinoma
ESPL1: extra spindle pole bodies like 1, separase
FOXM1: forkhead box M1
HDACi: histone deacetylase inhibitor
HR: homologous recombination
MASTL: microtubule associated serine/threonine kinase like
MSI-H: microsatellite instability-high
mu: mutant
PbCT: platinum-based chemotherapy
PCR: polymerase chain reaction
PFS: progression-free survival
PLK1: polo-like kinase 1
*POLE*-mu: polymerase ɛ mutant
PP2A: protein phosphatase 2A
TCGA: The Cancer Genome Atlas
USC: uterine serous carcinoma
wt: wild type
