# Supplementary figures and images for: Association of a novel endometrial cancer biomarker panel with prognostic risk, platinum insensitivity, and targetable therapeutic options
Source: PLoS One. 2021 Jan 27;16(1):e0245664. doi: 10.1371/journal.pone.0245664 (PMC7840025; doi:10.1371/journal.pone.0245664)

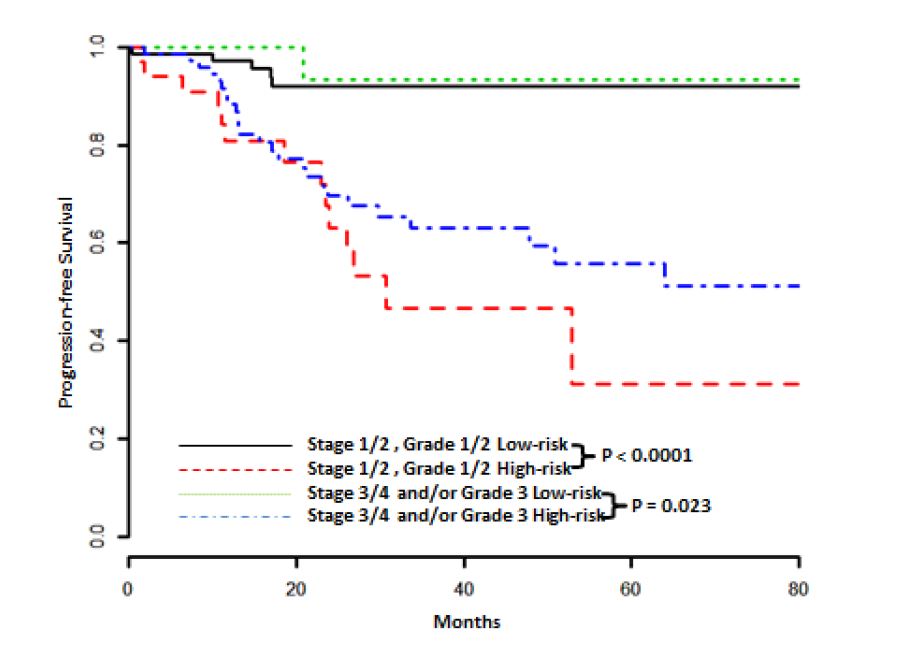

Supplement: S1 Fig — (JPG) [file pone.0245664.s001.JPG]

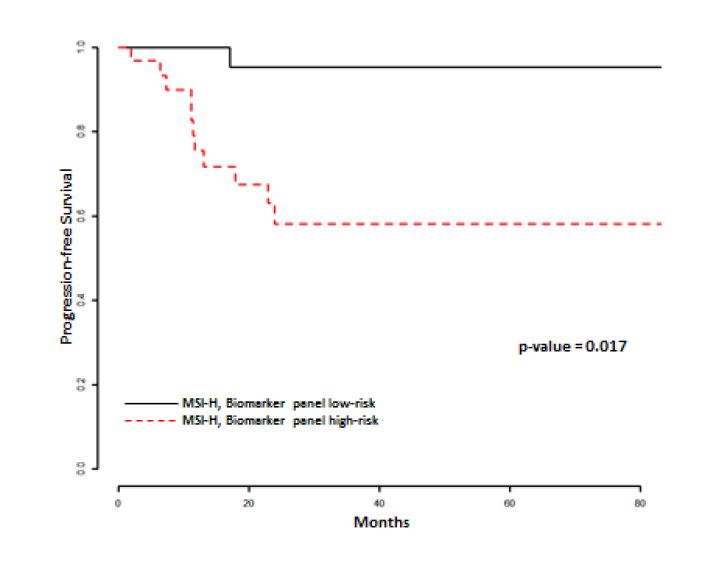

Supplement: S2 Fig — (JPG) [file pone.0245664.s002.JPG]

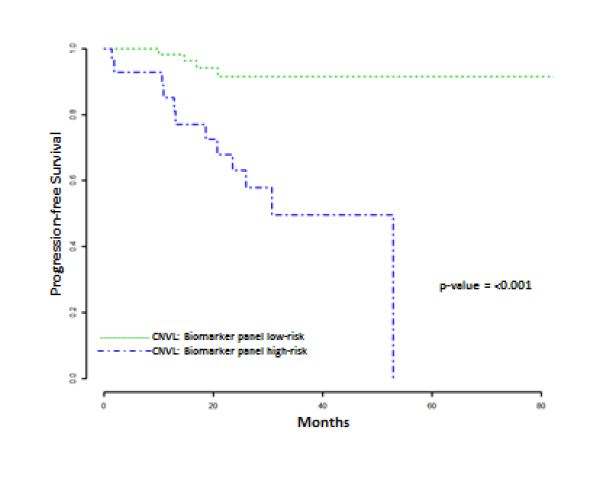

Supplement: S3 Fig — (JPG) [file pone.0245664.s003.JPG]
