# Supplementary material for: Association of a novel endometrial cancer biomarker panel with prognostic risk, platinum insensitivity, and targetable therapeutic options
Source: PLoS One. 2021 Jan 27;16(1):e0245664. doi: 10.1371/journal.pone.0245664 (PMC7840025; doi:10.1371/journal.pone.0245664)
Supplement: S1 Table — (DOCX) [file pone.0245664.s004.docx]

**Methods**

**S1 Table. PCR primer sequences.**

| Gene | Forward sequence (5'-3') | Reverse sequence (5'-3') |
| --- | --- | --- |
| *P21* | TGTCACTGTCTTGTACCCTTG | GGCGTTTGGAGTGGTAGAA |
| *P53* | GCCATCTACAAGCAGTCACAG | TCATCCAAATACTCCACACGC |
| *CCNA2* | CTGCATTTGGCTGTGAACTAC | ACAAACTCTGCTACTTCTGGG |
| *CCNE1* | TCTTGAGCAACACCCTCTTC | TTCTTGTGTCGCCATATACCG |
| *E2F1* | TCTCCGAGGACACTGACAG | ATCACCATAACCATCTGCTCTG |
| *CIP2A* | AGTCAGTACAAAGCCGTGAAG | ATAGTCGTGTGAGTTTCTGTCC |
| *EXO1* | GCCATAATTACAGAGGACTCGG | TTCCGTGAATACATCCCCAAG |
| *FOXM1* | ACCGCTACTTGACATTGGAC | GGGAGTTCGGTTTTGATGGTC |
| *GAPDH* | ACATCGCTCAGACACCATG | TGTAGTTGAGGTCAATGAAGGG |
