# Supplementary material for: Association of a novel endometrial cancer biomarker panel with prognostic risk, platinum insensitivity, and targetable therapeutic options
Source: PLoS One. 2021 Jan 27;16(1):e0245664. doi: 10.1371/journal.pone.0245664 (PMC7840025; doi:10.1371/journal.pone.0245664)
Supplement: S2 Table — (DOCX) [file pone.0245664.s005.docx]

**Results**

**S2 Table. Gene expressions as a function of molecular panel cohorts.**

| **Gene** | **mRNA Expression** | | | **Cohort A vs B** | | **Cohort A vs C** | |
| --- | --- | --- | --- | --- | --- | --- | --- |
|  | **Cohort A** | **Cohort B** | **Cohort C** | **Cohen’s**  **d^†^** | **P** | **Cohen’s**  **d^†^** | **P** |
|  | **Mean (SD)** | **Mean (SD)** | **Mean (SD)** |  |  |  |  |
| ***CIP2A*** | 0.934 (0.958) | 2.477 (0.882) | 1.567 (1.251) | 1.662 | <0.001 | 0.600 | 0.001 |
| ***FOXM1*** | 2.832 (0.721) | 4.340 (0.700) | 3.534 (0.898) | 2.112 | <0.001 | 0.903 | <0.001 |
| ***EXO1*** | 0.015 (1.039) | 1.437 (0.807) | 0.764 (0.918) | 1.494 | <0.001 | 0.746 | <0.001 |
| ***RAD51*** | 0.829 (0.775) | 2.138 (0.723) | 1.512 (0.950) | 1.735 | <0.001 | 0.822 | <0.001 |
| ***BRIP1*** | -1.109 (0.890) | 0.052 (0.915) | -0.643 (0.961) | 1.289 | <0.001 | 0.511 | 0.006 |
| ***BRCA1*** | 0.735 (0.778) | 1.665 (0.744) | 1.153 (0.941) | 1.216 | <0.001 | 0.504 | 0.007 |
| ***BRCA2*** | -2.236 (1.295) | -0.686 (1.106) | -1.546 (1.365) | 1.268 | <0.001 | 0.524 | 0.005 |
| ***SKP2*** | 1.926 (0.742) | 2.938 (0.738) | 2.276 (0.793) | 1.366 | <0.001 | 0.462 | 0.01 |
| ***MER11*** | 0.663 (0.752) | 1.200 (0.716) | 0.738 (0.862) | 0.728 | <0.001 | 0.096 | 0.60 |

†Cohen’s d = Absolute value of the difference in group means divided by the pooled standard deviation; the larger the number the

greater the difference between groups: ≥ 0.2 small, ≥0.5 medium and ≥0.8 large.
